# Associations of oral contraceptives with expression of CD44, CD24, and ALDH1A1 stem cell markers in women with benign breast biopsies

**DOI:** 10.1186/s13058-026-02310-y

**Published:** 2026-05-27

**Authors:** Lusine Yaghjyan, Yujing J. Heng, Brian R. Sardella, Maisey Ratcliffe, Bernard Rosner, Rulla M. Tamimi

**Affiliations:** 1 College of Public Health and Health Professions and College of Medicine, Department of Epidemiology, University of Florida, 2004 Mowry Rd., Gainesville, FL 32610 USA; 2 Department of Pathology, Beth Israel Deaconess Medical Center, Harvard Medical School, Boston, MA USA; 3 Channing Division of Network Medicine, Department of Medicine, Brigham and Women’s Hospital, Harvard Medical School, Boston, MA USA; 4 Department of Biostatistics, Harvard T.H. Chan School of Public Health, Boston, MA USA; 5 Department of Population Health Sciences, Weill Cornell Medicine, New York, NY USA

**Keywords:** Oral contraceptives, Stem cell markers, Benign breast disease, Automated image analysis

## Abstract

**Background:**

We investigated the association between oral contraceptives (OCs) and CD44, CD24, and ALDH1A1 stem cell (SC) markers’ expression in benign breast tissue.

**Methods:**

We included 731 cancer-free women with biopsy-confirmed benign breast disease (BBD) within the Nurses’ Health Study II. Information on OC use was collected in 1989 and updated biennially. Immunohistochemistry (IHC) was performed on tissue microarrays constructed from histologically normal breast tissue cores. For each core, the IHC expression was assessed with semi-automated software as the % of positively stained cells for each marker. Generalized linear regression was used to examine the associations of OCs (use status, total duration, age at first and last use, use before first pregnancy, and time since last use) with each marker’s log-transformed expression (within stroma and epithelium).

**Results:**

In multivariate analyses, OC use was positively associated with stromal CD44 expression (current vs. never β = 0.75, 95% Confidence Interval [CI] 0.11, 1.39, p-trend = 0.02). Age at first use was inversely associated with CD44 expression (in stroma: β= −0.04, 95% CI −0.08, −0.01; in epithelium β= −0.04, 95% CI −0.06, −0.02). Use before first pregnancy was positively associated with CD44 in epithelium (β = 0.43; 95% CI 0.13, 0.74). Total duration, age at last use, and time since last use were not associated with any of the SC markers.

**Conclusions:**

Our findings suggest a greater expression of CD44 in the breast stroma of current OC users and a reduced CD44 expression with a later age at first OC use, consistent with the reported associations of OCs with BCa risk.

**Supplementary Information:**

The online version contains supplementary material available at 10.1186/s13058-026-02310-y.

## Background

Recently, the role of stem cells in breast cancer development, progression, and treatment has been recognized as a high priority for translational breast cancer research [1]. Despite an important role of these stem cells in sustaining normal tissue architecture of the breast tissue throughout the woman’s life, potentially limitless proliferative and self-renewal capacity and high susceptibility to various endogenous and exogenous mutagenic insults increase the chances of their tumorigenic transformation [2, 3]. However, epidemiological studies linking known breast cancer risk factors with the expression of stem cell markers in normal breast tissue of cancer-free women remains very limited.

Oral contraceptives (OCs) have demonstrated to increase in breast cancer risk [4–9] with an earlier review of 44 studies demonstrating an 8% increased risk of breast cancer in OC users as compared to non-users (Odds Ratio [OR] = 1.08, 95% CI 1.00, 1.17) [4]. These associations were more apparent among current and recent users and were also more prominent among high-dose estrogen OC users [6–8]. It has been suggested that OCs can increase breast cancer risk by stimulating cellular proliferation and subsequently, increasing the risk of malignant transformation in the rapidly proliferating epithelium [10, 11]. Some studies have reported positive associations of OC use and inverse associations of the age at OC initiation with high mammographic breast density [12, 13], a well-established, strong breast cancer risk factor reflective of the proportion of fibroglandular tissue in the breast. On the other hand, mammographic breast density has been also recently linked to the expression of stem cell markers in cancer-free women [14] providing evidence in support of potential influence of OCs on breast stem cells. No study has examined the association of OCs with breast stem cell markers.

Well-characterized stem cell markers CD44 and CD24 have been linked to younger age at breast cancer diagnosis, more aggressive tumor characteristics, including triple-negative state, and metastasis [15–18]. Another stem cell marker, aldehyde dehydrogenase family 1 member A1 (ALDH1A1), is correlated with poor prognosis and chemotherapy resistant breast cancer [17, 19–23]. In this study, we examined the associations of the use of OCs with the expression of CD44, CD24, and ALDH1A1 markers in normal breast tissue of cancer-free women using prospective data from the Nurses’ Health Study II cohort. We hypothesized that OC use would be positively associated with the expression of CD44 and ALDH1A1 and inversely associated with CD24 (consistent with associations of CD44^high^, ALDH1A1^high^, and CD24^low^ with unfavorable tumor features) [15–23].

## Materials and methods

### Study population and design

Our study included women with incident biopsy-confirmed benign breast disease (BBD) from the NHSII. The NHSII included registered nurses across the US who were 25–42 years old at recruitment [24]. The initial 1989 NHSII questionnaire and all subsequent biennial questionnaires asked women to report whether they were diagnosed with fibrocystic or other BBD and whether the diagnosis was confirmed by biopsy or aspiration [25]. Cumulative response rates were > 90%, and response rates to each questionnaire were very similar among women with and without previously reported BBD [26]. Updated biennial questionnaires provided follow-up data on breast cancer risk factors and disease diagnoses, including BBD, confirmed through medical records [27]. Details about the study design methods for the NHSII and incident BBD study have been published previously [25, 27–29].

Women who reported a first diagnosis of biopsy-confirmed BBD were contacted to confirm the diagnosis and to acquire permission to review their pathology specimens. After permission was granted, benign breast biopsy slides were collected from hospital pathology departments and reviewed by study pathologists [25]. Exclusions were made for in situ or invasive carcinoma or unknown lesion type at biopsy. Our current analysis included 731 women with complete data on OC use and stem cell marker staining results. Distribution of breast cancer risk factors was similar in our sample vs. all women in the NHSII who had a benign breast biopsy so the chances for selection bias were minimal.

This study was conducted in accordance with the Declaration of Helsinki and was approved by the institutional review boards (IRBs) of Brigham and Women’s Hospital and Harvard T.H. Chan School of Public Health, alongside participating registries. Consent was either explicitly given or implied by the questionnaire return.

### Benign breast biopsy confirmation and BBD subtypes

Hematoxylin and eosin (H&E)-stained breast tissue slides from women with BBD underwent centralized pathological review and the pathologists were blinded to the original BBD diagnosis [30, 31]. Questionable or confirmed atypia cases were jointly reviewed. Each BBD case was classified as non-proliferative, proliferative without atypia, or atypical hyperplasia (ductal or lobular) according to standard categorization [26, 32].

### Tissue microarray (TMA) construction of BBD samples

After centralized review, formalin-fixed paraffin-embedded (FFPE) benign breast biopsy blocks were collected. H&E sections of corresponding tissue blocks were re-evaluated to annotate proliferative lesions and histologically normal terminal duct-lobular units (TDLUs). TMAs were constructed at the Dana Farber/Harvard Cancer Center (DF/HCC) Tissue Microarray Core Facility, and contained up to three 0.6 mm normal TDLU cores per woman. Our previously evaluated TMA construction method achieved a high success rate (76%) in capturing normal TDLUs [33].

### Immunohistochemistry (IHC) and image analysis for stem cell markers

For each of the three markers one 5-µm paraffin section was cut from a single TMA block and then stained with antibodies for CD44, CD24, and ALDH1A1 at the University of Florida Pathology Core Lab on DAKO AutostainerPlus using commercial antibodies (CD44 [DAKO, Carpinteria, CA] 1:25 dilution; CD24 [Invitrogen, Waltham, MA] 1:200 dilution and ALDH1A1 [Abcam, Cambridge, MA] 1:300 dilution). Details of this protocol have been described previously (9, 30–31). Briefly, slides were de-paraffinized with xylene and re-hydrated through decreasing concentrations of ethanol to water, including an intermediate step to quench endogenous peroxidase activity (3% hydrogen peroxide in methanol) and transferred to 1X TBS-T (Tris-buffered saline-Tween). For heat-induced antigen retrieval, sections were heated in a steamer while submerged in Citra (Biogenex, Fremont, CA) or Trilogy (Cell Marque, Rocklin, CA) for 30 minutes. Next, slides were 1) rinsed in 1X TBS-T and incubated with a universal protein blocker Sniper (Biocare Medical, Walnut Creek, CA) for 10 (for CD44 and ALDH1A1) or 15 minutes (for CD24); 2) rinsed in 1X TBS-T and co-incubated in primary antibody ALDH1A1, CD24, or CD44 for 1 hour; and 3) rinsed in 1X TBS-T followed by application of conjugated secondary antibody (Mach 2 goat anti-rabbit horse [or mouse] radish peroxidase-conjugated, Biocare Medical, Walnut Creek, CA) for 30 minutes. Detection of antibodies was achieved by incubating slides in 3’3’ diaminobenzidine (Vector Laboratories Inc., Burlingame, CA) for 4 min. Slides were counterstained with hematoxylin (Biocare Medical, Walnut Creek, CA) 1:10 for 3 min and mounted with Cytoseal XYL (Richard-Allen Scientific, Kalamazoo, MI). The core laboratory implemented standard quality control procedures.

Stained TMA sections were digitized at 20× using the PhenoImager HT (Akoya Biosciences, Marlborough, MA). QuPath v0.5.0 quantified the immunoreactivity of the markers [34]. For each marker, we randomly selected 12 tissue cores of variable staining intensities on a TMA to train tissue segmentation into epithelium, stroma, and background, and determine the minimum intensity to score a cell as immunoreactive [35]. We used random forest object classifier and the “positive cell detection” command to optimize cell parameters, intensity thresholds for hematoxylin and cytoplasm (mean optical density), and used the default values for all other parameters. We specifically focused on normal TDLU cores, due to their relevance to early breast carcinogenesis and high heterogeneity and smaller number of benign lesion cores. The staining was assessed separately in epithelium and stroma. In our previous work, we found high reliability of these markers. Briefly, spearman correlation between pathologist and Definiens readings ranged between 0.40 and 0.64 for stroma and 0.66–0.68 for epithelium. Our findings support the use of computational platforms for IHC evaluation of stem cell markers in large-scale epidemiologic studies [39].

### Assessment of oral contraceptive use

Details of oral contraceptive use assessment in NHSII were reported previously [24, 36]. Briefly, at baseline, women were asked to report if they had ever used OCs and if they were currently using OCs. For each year of age, women were asked to report if they had used OCs for at least 2 months in that year, if they had used OCs for at least 10 + months in that year, and the brand of OC used during that year. At each follow-up questionnaire cycle, women reported if they were currently using OCs, if they had used OCs since the last questionnaire, their duration of OC use since the last questionnaire in pre-specified categories (≤ 1, 2–4, 5–9, 10–14, 15–19, and ≥ 20 months), and the OC brand they had used for the longest during the last two years. To reduce recall inaccuracy, each questionnaire was accompanied with a booklet containing names and color photographs of all OC brands available during the relevant time period [36].

We assessed life-long OC exposure using all questionnaires available from before the biopsy date. The information on OC use was updated from the most recent questionnaire preceding the biopsy.

### Covariate information

Information on breast cancer risk factors was obtained from the biennial questionnaires closest to the date of the biopsy. The menopausal status of participants was determined using previously established criteria by considering their menstrual cycle cessation, oophorectomy, and hysterectomy [37, 38].

### Statistical analysis

We used generalized linear regression to examine the associations of oral contraceptive use with continuous expression (log-transformed) of each marker (separately for epithelium and stroma). We used a weighted average to summarize the marker expression level across available cores for a woman [39]. We first examined age and body mass index (BMI)-only adjusted models. Next, the risk estimates were adjusted for the following breast cancer risk factors: age (continuous), body mass index (BMI, continuous), age at menarche (< 12, 12, 13, > 13, unknown), combined parity/age at first birth (parous with first birth before age 25, parous with first birth at or after age 25, nulliparous, unknown), a family history of breast cancer (yes/no), menopausal status/postmenopausal hormone use (premenopausal, postmenopausal/no hormones, postmenopausal/past hormones, postmenopausal/current hormones, postmenopausal/unknown hormone use status), BBD subtype (non-proliferative, proliferative without atypia, proliferative with atypia), and alcohol use (none, > 0-<5, ≥ 5 g/day).

OC use was categorized with previously used approaches [8, 40] as follows: OC use status (never, past use, current use), duration of OC use (continuous [years] as well as categorical as: never, ≤ 1, 1 - <2, 2 - <5, 5-<10, and ≥ 10 years), time since last OC use (continuous [years] as well as categorical as: never, current, ≤ 4, >4 - <10, 10 - <15, and ≥ 15 years), age at first OC use (continuous [years] as well as categorical < 20, ≥20–24, 25–29, and ≥ 30 years), and age at last OC use (continuous [years] as well as categorical as: <20, 20–24, 25–29, and ≥ 30 years), and age at last use (continuous [years]). A two-sided test for trend was performed modeling relevant contraceptive use variables as ordinal variables and using the median level in each category. Based on our findings, we also examined mutually adjusted models that simultaneously included: (1) OC use status and age at first use; (2) OC status and duration of use; and (3) age at first use and duration of use. In sensitivity analysis, we examined these associations in premenopausal women only. Statistical significance in all the analyses was assessed at 0.05 level. The analyses were performed using SAS software (version 9.4, SAS Institute, Cary, NC, USA).

## Results

Of the 731 cancer-free women in the study, 106 (15%) were never, 577 (79%) were past, and 48 (8%) were current oral contraceptive users. The average age of the study population was 44 years (range 27–63). Most of the women were premenopausal (81%). Among women in the study, 27% had non-proliferative BBD, 65% had proliferative BBD without atypia, and 8% had proliferative BBD with atypia. The age-adjusted characteristics of the study population by OC use status are presented in Table 1. The expression of CD44 in epithelium and stroma appeared to be slightly higher among current OC users. The expression of CD24 and ALDH1A1 were similar across OC use subgroups.

**Table 1:** Age-adjusted characteristics of women with biopsy confirmed benign breast disease in the Nurses’ Health Study II, by oral contraceptive use

Tissue marker/tissue type	Oral contraceptive use		
	Never (*n* = 106)	Past (*n* = 577)	Current (*n* = 48)
*Mean (SD)*			
Age at BBD biopsy, years ^a^	43.52 (6.64)	44.82 (5.85)	40.65 (5.66)
Age at menarche	12.75 (1.31)	12.49 (1.37)	13.06 (1.39)
Body Mass Index, kg/m^2^	24.25 (4.71)	25.90 (6.00)	25.60 (5.00)
Alcohol use. g/day	3.66 (6.43)	3.94 (6.30)	4.71 (5.67)
CD44 in stroma	8.19 (8.67)	8.58 (9.00)	10.96 (8.43)
CD44 in epithelium	47.79 (25.11)	47.71 (23.94)	56.88 (22.25)
CD24 in stroma	43.31 (21.55)	43.23 (19.07)	42.55 (14.58)
CD24 in epithelium	74.28 (16.26)	72.86 (17.67)	72.48 (13.49)
ALDH1A1 in stroma	26.19 (19.87)	25.37 (20.79)	29.20 (17.70)
ALDH1A1 in epithelium	13.62 (9.57)	14.39 (12.28)	14.83 (8.66)
*Percentages*			
Parity/age at first birth			
Nulliparous	20	12	21
Parous/age < 25 years	21	32	18
Parous/age ≥ 25 years	59	57	61
Family history of breast cancer (yes)	6	10	7
Biopsy-confirmed benign breast disease			
Non-proliferative	26	26	28
Proliferative without atypia	64	66	63
Proliferative with atypia	10	8	8
Never smoked	77	64	63
Past smoker	16	25	37
Current smoker	7	11	0

^a^ No age adjustment

The results from age and BMI-only adjusted models are presented in Supplementary Table 1. In multivariable analyses (Table 2), OC use status was positively associated with the stromal expression of CD44 (current vs. never: β = 0.75, 95% CI 0.11, 1.39, *p*-trend = 0.02). Age at first use of OC was inversely associated with the expression of CD44 in both stromal (β per one year change = −0.04, 95% CI −0.08; −0.01; p-trend = 0.02) and epithelial regions (β per one year change=-0.04, 95% CI −0.06; −0.02); these associations appeared to be driven by the age group ≥ 30 which represented the smallest strata in our sample. Use before first pregnancy was positively associated with CD44 expression in epithelial regions (β = 0.43; 95% CI 0.13; 0.74). OC use status and age at first use were not associated with CD24 and ALDH1A1 expression. Total duration of use, time since last use, and age at last use were not associated with any of the SC markers (Table 2). The findings were similar in mutually adjusted models (data not shown). Finally, the associations were similar in the analysis limited to premenopausal women (Supplementary Table 2) though the association of age at first use with stromal CD44 did not reach statistical significance.

**Table 2:** Association of oral contraceptive (OC) use with expression of stem cell markers (log-transformed) in benign breast biopsy samples (β and 95% Confidence interval) ^a^

Exposure variable	CD44				CD24				ALDH1A1			
	N	Stroma	N	Epithelium	N	Stroma	N	Epithelium	N	Stroma	N	Epithelium
*OC Use Status*												
Never	105	Ref	103	Ref	103	Ref	101	Ref	104	Ref	103	Ref
Past	564	0.31 (−0.07; 0.70)	563	0.1 × 10^–2^ (−0.19; 0.20)	553	0.02 (−0.10; 0.14)	549	−0.04 (−0.11; 0.02)	552	−0.37 (−0.80; 0.07)	556	−0.09 (−0.33; 0.16)
Current	46	0.75 (0.11; 1.39)	46	0.24 (−0.08; 0.56)	46	0.07 (−0.13; 0.27)	46	−0.02 (−0.12; 0.08)	45	0.31 (−0.41; 1.03)	45	0.13 (−0.28; 0.53)
p-trend	715	0.02	712	0.28	702	0.53	696	0.39	701	0.93	704	0.87
*Total duration of use, yrs*												
Never	105	Ref	103	Ref	103	Ref	101	Ref	104	Ref	103	Ref
≤ 1	101	0.46 (−0.04; 0.96)	101	0.01 (−0.25; 0.26)	99	0.4 × 10^–2^ (−0.15; 0.16)	99	−0.04 (−0.12; 0.04)	99	−0.36 (−0.93; 0.21)	100	−0.14 (−0.46; 0.18)
> 1—< 2	35	0.40 (−0.29; 1.10)	35	0.05 (−0.31; 0.40)	35	−0.04 (−0.26; 0.17)	35	−0.09 (−0.20; 0.02)	36	−0.12 (−0.90; 0.67)	36	−0.06 (−0.49; 0.38)
2—< 5	185	0.35 (−0.09; 0.78)	185	0.02 (−0.21; 0.24)	183	0.02 (−0.12; 0.16)	180	−0.05 (−0.12; 0.02)	181	−0.53 (−1.04; −0.03)	181	−0.04 (−0.33; 0.24)
5—< 10	190	0.28 (−0.16; 0.71)	189	−0.05 (−0.27; 0.17)	185	0.06 (−0.08; 0.20)	184	−0.01 (−0.08; 0.06)	187	−0.36 (−0.86; 0.14)	186	−0.06 (−0.34; 0.22)
≥ 10	83	0.36 (−0.17; 0.88)	83	0.16 (−0.11; 0.42)	81	0.05 (−0.11; 0.22)	81	−0.03 (−0.12; 0.05)	79	0.16 (−0.44; 0.77)	82	−0.06 (−0.40; 0.28)
p-trend	699	0.64	696	0.5	686	0.28	680	0.83	686	0.41	688	0.99
Total duration of use, yrs	699	0.02 (−0.01; 0.05)	696	0.01 (−0.01; 0.03)	686	0.5 × 10^–2^ (−0.5 × 10^–2^; 0.01)	680	−0.1 × 10^–3^(−0.01; 0.5 × 10^–2^)	686	0.02 (−0.02; 0.05)	688	0.3 × 10^–3^ (−0.02; 0.02)
*Years since last use*												
Never	105	Ref	103	Ref	103	Ref	101	Ref	104	Ref	103	Ref
Current	46	0.74 (0.10; 1.39)	46	0.23 (−0.09; 0.56)	46	0.07 (−0.13; 0.27)	46	−0.03 (−0.13; 0.08)	45	0.29 (−0.43; 1.01)	45	0.12 (−0.28; 0.53)
≤ 4	69	0.17 (−0.39; 0.73)	68	0.08 (−0.20; 0.36)	67	−0.09 (−0.27; 0.08)	65	−0.08 (−0.17; 0.01)	67	−0.14 (−0.77; 0.49)	68	−0.20 (−0.55; 0.15)
> 4—< 10	116	0.14 (−0.35; 0.63)	116	−0.17 (−0.42; 0.07)	113	−0.03 (−0.18; 0.12)	112	−0.09 (−0.17; −0.01)	114	−0.51 (−1.06; 0.04)	115	−0.25 (−0.55; 0.06)
10—< 15	101	0.34 (−0.16; 0.85)	101	−0.04 (−0.29; 0.21)	99	0.08 (−0.07; 0.24)	99	−0.04 (−0.12; 0.04)	99	−0.62 (−1.19; −0.06)	98	−0.01 (−0.32; 0.31)
≥ 15	274	0.42 (−0.01; 0.85)	274	0.08 (−0.14; 0.29)	270	0.05 (−0.08; 0.19)	269	−0.01 (−0.08; 0.06)	268	−0.25 (−0.73; 0.23)	271	0.00 (−0.27; 0.27)
p−trend	711	0.15	708	0.14	698	0.21	692	0.03	697	0.83	700	0.12
Years since last use	560	0.02 (−0.3 × 10^–2^, 0.04)	559	0.01 (−0.2 × 10^–2^; 0.02)	549	0.4 × 10^–2^ (−0.3 × 10^–2^; 0.01)	545	0.3 × 10^–2^ (−0.4 × 10^–3^; 0.01)	548	0.3 × 10^–2^ (−0.02; 0.03)	552	0.01 (−0.3 × 10^–2^; 0.02)
*Age at 1st use, yrs*												
< 20	262	Ref	261	Ref	258	Ref	256	Ref	258	Ref	260	Ref
20–24	262	−0.41 (−0.70; −0.12)	262	−0.22 (−0.38; −0.05)	258	0.03 (−0.06; 0.13)	256	−0.01 (−0.06; 0.04)	258	−0.37 (−0.75; 0.01)	258	−0.12 (−0.32; 0.09)
25–29	43	−0.41 (−0.96; 0.13)	43	−0.18 (−0.49; 0.13)	41	0.16 (−0.02; 0.34)	41	0.06 (−0.03; 0.16)	41	0.51 (−0.22; 1.24)	42	0.20 (−0.19; 0.59)
≥ 30	23	−0.66 (−1.38; 0.06)	23	−0.84 (−1.25; −0.43)	22	0.07 (−0.17; 0.30)	22	0.8 × 10^–3^ (−0.13; 0.13)	21	−0.36 (−1.34; 0.62)	21	−0.60 (−1.13; −0.07)
p-trend	590	0.02	589	< 0.0001	579	0.19	575	0.57	578	0.87	581	0.16
Age at 1st use, yrs	590	−0.04 (−0.08; −0.01)	589	-0.04 (−0.06; −0.02)	579	0.01 (−0.2 × 10^–2^; 0.02)	575	0.6 × 10^–3^ (−0.01; 0.01)	578	−0.01 (−0.06; 0.03)	581	−0.02 (−0.04; 0.4 × 10^–2^)
Age at last use, yrs	540	−0.01 (−0.03; 0.01)	539	−0.01 (−0.02; 0.4 × 10^–2^)	529	−0.3 × 10^–2^(−0.01; 0.3 × 10^–2^)	525	−0.3 × 10^–2^(−0.01; 0.4 × 10^–3^)	529	−0.4 × 10^–2^ (−0.03; 0.02)	532	−0.01 (−0.03; 0.3 × 10^–2^)
*Use before 1st pregnancy*												
No	48	Ref	48	Ref	47	Ref	47	Ref	47	Ref	47	Ref
Yes	468	0.05 (−0.47; 0.58)	468	0.43 (0.13; 0.74)	461	−0.06 (−0.22; 0.11)	458	0.3 × 10^–2^ (−0.09; 0.09)	459	−0.10 (−0.75; 0.54)	462	0.06 (−0.32; 0.43)

^a^ Adjusted for age (continuous), BMI (continuous), a family history of breast cancer (Yes/No), menopausal status/postmenopausal hormone use (premenopausal, postmenopausal/no hormones, postmenopausal/past hormones, postmenopausal/current hormones, postmenopausal/unknown hormone use status), age at menarche (< 12, 12, 13, > 13, unknown), combined parity/age at first birth (parous with first birth before age 25, parous with first birth at or after age 25, nulliparous, unknown), BBD category (non-proliferative, proliferative without atypia, and proliferative with atypia), and NHS cohort (NHSI, NHSII)

## Discussion

In this study, we investigated the associations of OC use the expression of CD44, CD24, and ALDH1A1 stem cell markers in benign breast biopsy samples of cancer-free women. We found positive associations of OC status and inverse associations of age at first use with CD44 expression.

We provide the first evidence of the association between exposure to OCs and the expression of stem cell markers in normal breast tissue. OC use has been reported to increase breast cancer risk, including a recent review of 44 studies with stronger associations in current and recent users [4–9]. Some of the potential mechanisms underlying these associations include increase in cellular proliferation which may lead to increased chances of tumor initiation is rapidly dividing epithelium [10, 11]. In line with this mechanism, some previous studies have reported positive associations of OC use and inverse associations of the age at OC initiation with high breast density [12, 13]. On the other hand, mammographic breast density has been also recently linked to the expression of stem cell markers in cancer-free women [14, 41] suggesting that OCs may potentially also affect breast stem cells. While no studies have examined the associations of oral contraceptives with breast stem cell markers, our findings along with the presented evidence collectively suggests that OCs may influence breast stem cells, leading to an increase in breast density and subsequent breast cancer risk.

Our results also indicated that women who start using OCs at younger age as well as those who used OCs before first pregnancy have greater expression of CD44. Before the first full-term pregnancy, the undifferentiated breast tissue remains highly susceptible to various endogenous and exogenous carcinogenic influences that could later be reflected in adult breast density and breast cancer risk [42–47]. Additionally, pregnancy is further believed to downregulate signaling pathways known to play a role in mammary stem cell function [48] thus leading to a decrease in stem cell pool size and activity [49, 50]. Earlier initiation of OCs in this window of susceptibility might result in more prominent effects of these exogenous hormones on the breast tissue which coupled with subsequent delay in the age at first pregnancy could delay these changes in stem cell pool leading to a greater expression of stem cell markers later in life and potentially, a greater risk of subsequent breast cancer. However, future studies are needed to confirm our findings and to elucidate underlying biological mechanisms. Finally, despite our findings for age at first use, we did not observe any associations for duration of use which could be explained by a weak correlation of these two variables in our sample (correlation coefficient = 0.24).

Our study is the first study to investigate the association of OCs with the expression of stem cell markers in cancer-free women with benign breast biopsies. The analysis used data from the NHSII, an established cohort with more than 25 years of follow-up, ascertainment of disease status and comprehensive information on breast cancer risk factors. A few limitations should be noted. Despite the prospective nature of the cohort, potential errors in OC use recall are possible; however, previous validation studies in this cohort demonstrated high accuracy in the self-report of different aspects of OC use [51]. While the biopsy samples were obtained from specific regions of the breast, our previous studies have indicated that this tissue sampling approach still provides robust evidence for measuring breast tissue involution [52], for identification of breast cancer biomarkers [53–55], and for establishing the correlation with various breast cancer risk factors [56]. Next, our study included only cancer-free women with benign breast biopsies potentially limiting generalizability of the findings; however, given our specific focus on normal TDLUs, we believe the results could still apply to a broader spectrum of women without BBD [57]. Finally, in our study we did not use co-localization techniques for the IHC staining, which precluded us from evaluating the combined expression of these markers at the individual cell level [58].

## Conclusions

We investigated the associations of OC use with stem cell markers in normal TDLUs of cancer-free women. Our results suggest that current OC users as well as women with earlier age at initiation of OC use and OC use before first pregnancy could have greater expression of CD44. If confirmed, these findings could suggest an importance of limiting early-life OC exposure for breast cancer risk prevention. Further studies are warranted to confirm our findings.

## Supplementary Information

Below is the link to the electronic supplementary material.

Supplementary Material 1

## Data Availability

The data that support the findings of this study are available from the Nurses’ Health Studies, however they are not publicly available. Investigators interested in using the data can request access, and feasibility will be discussed at an investigators’ meeting. Limits are not placed on scientific questions or methods, and there is no requirement for co-authorship. Additional data sharing information and policy details can be accessed at http://www.nurseshealthstudy.org/researchers.

## Abbreviations

BBD: Benign breast disease
BMI: Body mass index
CI: Confidence interval
FFPE: Formalin-fixed paraffin-embedded
NHSII: Nurses’ Health Study II
OC: Oral contraceptives
TDLU: Terminal duct-lobular units
TMA: Tissue microarray
