# Supplementary material for: Associations of oral contraceptives with expression of CD44, CD24, and ALDH1A1 stem cell markers in women with benign breast biopsies
Source: Breast Cancer Res. 2026 May 27;28:135. doi: 10.1186/s13058-026-02310-y (PMC13397646; doi:10.1186/s13058-026-02310-y)
Supplement: Supplementary file 1 — Supplementary Material 1 [file 13058_2026_2310_MOESM1_ESM.docx]

**Supplementary table 1. Age and BMI-adjusted associations of oral contraceptive (OC) use with log-transformed expression of stem cell markers in benign breast biopsy samples**

| **Exposure variable** | **CD44** | | | | **CD24** | | | | **ALDH1A1** | | | |  |
| --- | --- | --- | --- | --- | --- | --- | --- | --- | --- | --- | --- | --- | --- |
|  | **N** | **Stroma** | **N** | **Epithelium** | **N** | **Stroma** | **N** | **Epithelium** | **N** | **Stroma** | **N** | **Epithelium** | |
| **OC Use Status**  Never  Past  Current  p-trend | 105  572  48  725 | Ref  0.32 (-0.06; 0.71)  0.81 (0.17; 1.44)  0.01 | 103  571  48  722 | Ref  -0.01 (-0.20; 0.19)  0.25 (-0.07; 0.56)  0.27 | 103  560  48  711 | Ref  0.03 (-0.09; 0.15)  0.05 (-0.15; 0.24)  0.59 | 101  556  48  705 | Ref  -0.03 (-0.09; 0.03)  -0.02 (-0.12; 0.08)  0.47 | 104  560  47  711 | Ref  -0.34 (-0.77; 0.09)  0.28 (-0.43; 0.98)  0.96 | 103  564  47  714 | Ref  -0.08 (-0.32; 0.16)  0.11 (-0.29; 0.51)  0.90 | |
| **Total duration of use, yrs**  Never  ≤ 1  >1 - <2  2 - <5  5 - <10  ≥ 10  p-trend | 105  103  35  187  194  85  709 | Ref  0.50 (0.01; 0.99)  0.45 (-0.24; 1.15)  0.35 (-0.08; 0.78)  0.28 (-0.15; 0.71)  0.41 (-0.11; 0.93)  0.61 | 103  103  35  187  193  85  706 | Ref  0.01 (-0.24; 0.26)  0.08 (-0.27; 0.43)  0.5x10^-2^ (-0.22; 0.23)  -0.06 (-0.28; 0.16)  0.16 (-0.10; 0.43)  0.51 | 103  101  35  184  189  83  695 | Ref  0.03 (-0.12; 0.19)  -0.06 (-0.27; 0.16)  0.03 (-0.10; 0.17)  0.06 (-0.07; 0.20)  0.04 (-0.12; 0.20)  0.46 | 101  101  35  181  188  83  689 | Ref  -0.03 (-0.10; 0.05)  -0.08 (-0.19; 0.03)  -0.04 (-0.11; 0.03)  -0.4x10^-2^ (-0.07; 0.06)  -0.04 (-0.12; 0.04)  0.99 | 104  101  36  183  191  81  696 | Ref  -0.29 (-0.85; 0.27)  -0.14 (-0.92; 0.64)  -0.49 (-0.98; 0.01)  -0.33 (-0.82; 0.16)  0.14 (-0.46; 0.74)  0.45 | 103  102  36  183  190  84  698 | Ref  -0.12 (-0.44; 0.20)  -0.06 (-0.50; 0.38)  -0.03 (-0.31; 0.25)  -0.06 (-0.33; 0.22)  -0.08 (-0.42; 0.25)  0.84 | |
| **Total duration of use, yrs** | 709 | 0.02 (-0.01; 0.05) | 706 | 0.01 (-0.01; 0.02) | 695 | 0.3x10^-2^ (-0.01; 0.01) | 689 | 0.1x10^-2^ (-0.01; 0.4x10^-2^) | 696 | 0.02 (-0.02; 0.05) | 698 | -0.1x10^-2^ (-0.02; 0.02) | |
| **Years since last use**  Never  Current  ≤4  >4 - <10  10 - <15  ≥15  p-trend | 105  48  70  117  103  278  721 | Ref  0.80 (0.16; 1.43)  0.18 (-0.38; 0.74)  0.15 (-0.33; 0.64)  0.38 (-0.12; 0.88)  0.41 (-0.02; 0.83)  0.16 | 103  48  69  117  103  278  718 | Ref  0.24 (-0.08; 0.56)  0.08 (-0.20; 0.35)  -0.17 (-0.41; 0.07)  -0.05 (-0.30; 0.20)  0.06 (-0.16; 0.27)  0.19 | 103  48  68  114  101  273  707 | Ref  0.04 (-0.16; 0.24)  -0.10 (-0.27; 0.08)  -0.04 (-0.19; 0.12)  0.09 (-0.07; 0.25)  0.07 (-0.06; 0.21)  0.05 | 101  48  66  113  101  272  701 | Ref  -0.03 (-0.13; 0.07)  -0.07 (-0.16; 0.01)  -0.09 (-0.16; -0.01)  -0.03 (-0.11; 0.05)  0.4x10^-3^ (-0.07; 0.07)  0.01 | 104  47  68  115  101  272  707 | Ref  0.27 (-0.44; 0.97)  -0.16 (-0.78; 0.47)  -0.48 (-1.02; 0.06)  -0.58 (-1.13; -0.02)  -0.23 (-0.70; 0.24)  0.66 | 103  47  69  116  100  275  710 | Ref  0.10 (-0.30; 0.49)  -0.22 (-0.57; 0.13)  -0.27 (-0.57; 0.03)  0.02 (-0.29; 0.34)  0.01 (-0.26; 0.27)  0.05 | |
| **Years since last use** | 568 | 0.02 (-0.3x10^-2^; 0.04) | 567 | 0.01 (-0.3x10^-2^; 0.02) | 556 | 0.01 (-0.1x10^-2^; 0.01) | 552 | 0.4x10^-2^ (0.4x10^-3^; 0.01) | 556 | 0.01 (-0.02; 0.03) | 560 | 0.01 (0.1x10^-3^; 0.03) | |
| **Age at 1^st^ use, yrs**  <20  20-24  25-29  ≥30  p-trend | 267  266  45  23  601 | Ref  -0.39 (-0.67; -0.11)  -0.38 (-0.91; 0.15)  -0.64 (-1.35; 0.07)  0.02 | 266  266  45  23  600 | Ref  -0.20 (-0.36; -0.04)  -0.15 (-0.44; 0.15)  -0.80 (-1.20; -0.40)  0.0001 | 262  262  43  22  589 | Ref   - 1. (-0.08; 0.11)   0.10 (-0.07; 0.28)  -0.01 (-0.24; 0.23)  0.60 | 260  260  43  22  585 | Ref  -0.01 (-0.06; 0.03)  0.04 (-0.05; 0.13)  -0.01 (-0.13; 0.12)  0.81 | 263  262  43  21  589 | Ref  -0.39 (-0.76; -0.02)  0.38 (-0.32; 1.08)  -0.41 (-1.38; 0.55)  0.66 | 265  262  44  21  592 | Ref  -0.15 (-0.35; 0.05)  0.13 (-0.24; 0.51)  -0.67 (-1.19; -0.15)  0.07 | |
| **Age at 1^st^ use, yrs** | 601 | -0.04 (-0.08; -0.01) | 600 | -0.04 (-0.06; -0.02) | 589 | 0.4x10^-2^ (-0.01; 0.02) | 585 | -0.2x10^-3^ (-0.01; 0.01) | 589 | -0.02 (-0.06; 0.03) | 592 | -0.02(-0.05; -0.3x10^-3^) | |
| **Age at last use, yrs** | 548 | -0.02 (-0.04; 0.3x10^-2^) | 547 | -0.01 (-0.02; 0.3x10^-2^) | 536 | -0.01 (-0.01; 0.1x10^-2^) | 532 | -0.4x10^-2^ (-0.01; -0.3x10^-3^) | 537 | -0.01 (-0.03; 0.02) | 540 | -0.01(-0.03; -0.3x10^-3^) | |
| **Use before 1^st^ pregnancy**  No  Yes | 48  468 | Ref  -0.02 (-0.53; 0.48) | 48  468 | Ref  0.39 (0.10; 0.68) | 47  461 | Ref  -0.12 (-0.28; 0.04) | 47  458 | Ref  -0.01 (-0.09; 0.08) | 47  459 | Ref  -0.24 (-0.86; 0.39) | 47  462 | Ref  -0.06 (-0.43; 0.30) | |

**Supplementary table 2. Association of oral contraceptive (OC) use with expression of stem cell markers (log-transformed) in benign breast biopsy samples in premenopausal women (β and 95% Confidence interval) ^a^**

| **Exposure variable** | **CD44** | | | | **CD24** | | | | **ALDH1A1** | | | |
| --- | --- | --- | --- | --- | --- | --- | --- | --- | --- | --- | --- | --- |
|  | **N** | **Stroma** | **N** | **Epithelium** | **N** | **Stroma** | **N** | **Epithelium** | **N** | **Stroma** | **N** | **Epithelium** |
| **OC Use Status**  Never  Past  Current  p-trend | 88  450  43  581 | Ref  0.26 (-0.16; 0.69)  0.69 (0.01; 1.37)  0.05 | 87  449  43  579 | Ref  0.1x10^-2^ (-0.21; 0.22)  0.21 (-0.13; 0.55)  0.35 | 86  441  43  570 | Ref  0.01 (-0.12; 0.14)  0.05 (-0.16; 0.26)  0.68 | 85  438  43  566 | Ref  -0.04 (-0.11; 0.03)  -0.03 (-0.14; 0.08)  0.41 | 87  440  42  569 | Ref  -0.27 (-0.74; 0.20)  0.40 (-0.36; 1.16)  0.72 | 87  443  42  572 | Ref  -0.1x10^-2^ (-0.26; 0.25)  0.17 (-0.24; 0.58)  0.53 |
| **Total duration of use, yrs**  Never  ≤ 1  >1 - <2  2 - <5  5 - <10  ≥ 10  p-trend | 88  86  31  150  149  65  569 | Ref  0.41 (-0.13; 0.96)  0.51 (-0.24; 1.26)  0.39 (-0.09; 0.87)  0.14 (-0.33; 0.62)  0.27 (-0.32; 0.85)  0.85 | 87  86  31  150  148  65  567 | Ref  -0.04 (-0.31; 0.24)  0.14 (-0.24; 0.52)  0.06 (-0.19; 0.30)  -0.06 (-0.30; 0.19)  0.13 (-0.17; 0.43)  0.72 | 86  85  30  149  144  64  558 | Ref  -0.02 (-0.18; 0.14)  0.10 (-0.13; 0.33)  0.02 (-0.12; 0.17)  0.02 (-0.13; 0.16)  0.03 (-0.15; 0.20)  0.77 | 85  85  30  147  143  64  554 | Ref  -0.05 (-0.13; 0.04)  -0.02 (-0.14; 0.09)  -0.04 (-0.12; 0.03)  -0.01 (-0.09; 0.06)  -0.04 (-0.13; 0.05)  0.91 | 87  84  31  148  146  62  558 | Ref  -0.32 (-0.94; 0.30)  0.22 (-0.63; 1.07)  -0.40 (-0.95; 0.15)  -0.27 (-0.82; 0.27)  0.27 (-0.40; 0.95)  0.41 | 87  85  31  148  145  64  560 | Ref  -0.08 (-0.42; 0.25)  0.06 (-0.40; 0.52)  0.03 (-0.26; 0.33)  0.03 (-0.26; 0.33)  0.03 (-0.33; 0.40)  0.68 |
| **Total duration of use, yrs** | 569 | 0.01 (-0.03; 0.04) | 567 | 0.01 (-0.01; 0.02) | 558 | 0.2x10^-2^ (-0.01; 0.01) | 554 | -0.2x10^-2^ (-0.01; 0.4x10^-2^) | 558 | 0.02 (-0.02; 0.06) | 560 | 0.01 (-0.01; 0.03) |
| **Years since last use**  Never  Current  ≤4  >4 - <10  10 - <15  ≥15  p-trend | 88  43  59  101  88  199  578 | Ref  0.68 (0.2x10^-2^; 1.37)  0.17 (-0.44; 0.78)  0.14 (-0.39; 0.67)  0.30 (-0.25; 0.85)  0.34 (-0.14; 0.82)  0.30 | 87  43  58  101  88  199  576 | Ref  0.21 (-0.13; 0.55)  0.07 (-0.24; 0.37)  -0.13 (-0.40; 0.14)  -0.04 (-0.32; 0.23)  0.07 (-0.17; 0.31)  0.28 | 86  43  58  98  86  196  567 | Ref  0.05 (-0.16; 0.25)  -0.07 (-0.26; 0.12)  -0.08 (-0.24; 0.08)  0.11 (-0.06; 0.28)  0.03 (-0.12; 0.18)  0.29 | 85  43  56  98  86  195  563 | Ref  -0.03 (-0.14; 0.07)  -0.07 (-0.16; 0.03)  -0.11 (-0.19; -0.03)  -0.02 (-0.11; 0.07)  -0.4x10^-3^ (-0.08; 0.08)  0.03 | 87  42  58  99  86  194  566 | Ref  0.39 (-0.37; 1.15)  0.06 (-0.62; 0.74)  -0.24 (-0.83; 0.35)  -0.59 (-1.20; 0.02)  -0.24 (-0.78; 0.29)  0.37 | 87  42  58  100  85  197  569 | Ref  0.17 (-0.24; 0.58)  -0.05 (-0.42; 0.32)  -0.12 (-0.44; 0.20)  0.09 (-0.24; 0.43)  0.04 (-0.25; 0.33)  0.64 |
| **Years since last use** | 447 | 0.02 (-0.01; 0.04) | 446 | 0.01 (-0.01; 0.02) | 438 | 0.3x10^-2^ (-0.4x10^-2^; 0.01) | 435 | 0.4x10^-2^ (-0.1x10^-3^; 0.01) | 437 | -0.02 (-0.05; 0.01) | 440 | 0.3x10^-2^ (-0.01; 0.02) |
| **Age at 1^st^ use, yrs**  <20  20-24  25-29  ≥30  p-trend | 201  216  41  19  477 | Ref  -0.44 (-0.77; -0.11)  -0.13 (-0.70; 0.45)  -0.51 (-1.32; 0.29)  0.18 | 200  216  41  19  476 | Ref  -0.19 (-0.37; 0.2x10^-2^)  -0.10 (-0.43; 0.22)  -0.77 (-1.23; -0.32)  0.003 | 198  212  40  18  468 | Ref  0.05 (-0.05; 0.15)  0.19 (0.02; 0.37)  0.03 (-0.21; 0.28)  0.19 | 197  210  40  18  465 | Ref   - 1. (-0.05; 0.06)   0.08 (-0.02; 0.18)  -0.01 (-0.15; 0.13)  0.48 | 197  212  40  18  467 | Ref  -0.40 (-0.83; 0.03)  0.54 (-0.21; 1.29)  -0.29 (-1.35; 0.77)  0.88 | 199  212  40  18  469 | Ref  -0.11 (-0.34; 0.11)  0.31 (-0.08; 0.69)  -0.58 (-1.13; -0.03)  0.36 |
| **Age at 1^st^ use, yrs** | 477 | -0.04 (-0.08; 0.2x10^-2^) | 476 | -0.04 (-0.06; -0.01) | 468 | 0.01 (-0.01; 0.02) | 465 | 0.00 (-0.01; 0.01) | 467 | -0.01 (-0.06; 0.04) | 469 | -0.02 (-0.05; 0.01) |
| **Age at last use, yrs** | 432 | -0.01 (-0.04; 0.01) | 431 | -0.01 (-0.02; 0.01) | 423 | -0.3x10^-2^(-0.01; 0.4x10^-2^) | 420 | -0.4x10^-2^ (-0.01; 0.4x10^-3^) | 423 | 0.02 (-0.01; 0.05) | 425 | -0.4x10^-2^ (-0.02; 0.01) |
| **Use before 1^st^ pregnancy**  No  Yes | 32  377 | Ref  0.11 (-0.51; 0.73) | 32  377 | Ref  0.49 (0.12; 0.86) | 31  372 | Ref  -0.08 (-0.27; 0.11) | 31  370 | Ref  0.03 (-0.08; 0.13) | 31  370 | Ref  0.11 (-0.64; 0.86) | 31  372 | Ref  0.23 (-0.20; 0.66) |

^a^ Adjusted for age (continuous), BMI (continuous), a family history of breast cancer (Yes/No), age at menarche (<12, 12, 13, >13, unknown), combined parity/age at first birth (parous with first birth before age 25, parous with first birth at or after age 25, nulliparous, unknown), BBD category (non-proliferative, proliferative without atypia, and proliferative with atypia), and NHS cohort (NHSI, NHSII).
